# The Nutritional and Pharmacological Potential of New Australian Thraustochytrids Isolated from Mangrove Sediments

**DOI:** 10.3390/md18030151

**Published:** 2020-03-06

**Authors:** Thi Linh Nham Tran, Ana F. Miranda, Adarsha Gupta, Munish Puri, Andrew S. Ball, Benu Adhikari, Aidyn Mouradov

**Affiliations:** 1 School of Sciences, Royal Melbourne Institute of Technology University, 3083 Bundoora, Australia; s3654084@student.rmit.edu.au (T.L.N.T.); ana.miranda@rmit.edu.au (A.F.M.); andy.ball@rmit.edu.au (A.S.B.); benu.adhikari@rmit.edu.au (B.A.); 2 Centre for Marine Bioproducts Development, College of Medicine and Public Health, Flinders University, 5042 Adelaide, Australia; adarsha.gupta@flinders.edu.au (A.G.); munish.puri@flinders.edu.au (M.P.)

**Keywords:** *Aurantiochytrium*, carbohydrates, carotenoids, EPS, FAME, lipids, protists, oil body, squalene

## Abstract

Mangrove sediments represent unique microbial ecosystems that act as a buffer zone, biogeochemically recycling marine waste into nutrient-rich depositions for marine and terrestrial species. Marine unicellular protists, thraustochytrids, colonizing mangrove sediments have received attention due to their ability to produce large amounts of long-chain ω3-polyunsaturated fatty acids. This paper represents a comprehensive study of two new thraustochytrids for their production of valuable biomolecules in biomass, de-oiled cakes, supernatants, extracellular polysaccharide matrixes, and recovered oil bodies. Extracted lipids (up to 40% of DW) rich in polyunsaturated fatty acids (up to 80% of total fatty acids) were mainly represented by docosahexaenoic acid (75% of polyunsaturated fatty acids). Cells also showed accumulation of squalene (up to 13 mg/g DW) and carotenoids (up to 72 µg/g DW represented by astaxanthin, canthaxanthin, echinenone, and β-carotene). Both strains showed a high concentration of protein in biomass (29% DW) and supernatants (2.7 g/L) as part of extracellular polysaccharide matrixes. Alkalinization of collected biomass represents a new and easy way to recover lipid-rich oil bodies in the form of an aqueous emulsion. The ability to produce added-value molecules makes thraustochytrids an important alternative to microalgae and plants dominating in the food, pharmacological, nutraceutical, and cosmetics industries.

## 1. Introduction

Global climate change, increasing population, and extensive use of arable lands are making our agriculture and related food and pharmacological industries unsustainable. This triggered an unprecedented search for the domestication of the next generation of renewable feedstock which can produce the main food components-protein, carbohydrates, healthy oils, antioxidants, and vitamins-under climate-independent conditions, such as bioreactors. Mangrove ecosystems represent unique microbial communities that act as a buffer, recycling marine waste into nutrient-rich depositions for marine species (fish, crab, shrimp, and mollusk) and terrestrial plants. Marine unicellular, heterotrophic protists, thraustochytrids, colonizing mangrove sediments and leaves have received attention due to their ability to produce large amounts of long-chain omega-3 polyunsaturated fatty acids (PUFAs) [1,2]. Thraustochytrids comprise nine genera with *Thraustochytrium*, *Aurantiochytrium*, *Schizochytrium*, and *Ulkenia* being the most investigated genera.

Fatty acid synthesis in thraustochytrids is more complex than in photosynthetic species, such as cyanobacteria, microalgae, and plants, and contains two independent pathways. A type I fatty acid synthase pathway producing the C16 type of fatty acids is similar to the one found in plants and mammals and synthesizes a saturated version of C16 fatty acids, palmitic acid (C16:0), which is a well-known feedstock for biodiesel production. The second pathway involves the production of very-long-chain ω3- and ω6-polyunsaturated fatty acids (VLC-PUFAs), such as eicosapentaenoic acid (EPA, C20:5), docosapentaenoic acid (DPA, C22:5), and docosahexaenoic acid (DHA, C22:6) [3]. The oil from *Schizochytrium* sp. is one of the most analyzed and tested microbial oils, and it has been designated as Generally Recognized as Safe by the U.S. Food and Drug Administration (FDA), being completely safe for human as well as animal consumption [4]. One liter of the fed-batch-grown *Aurantiochytrium* sp. can produce 12 g of dry biomass containing 1.6 g of DHA within five days [2,5]. This is equivalent to the amount of DHA accumulated in 500 g of Atlantic salmon (https://sciencing.com/amount-epa-dha-salmon-7435.html). DHA plays a significant role in enhancing human health and preventing human diseases, such as atherosclerosis, rheumatoid arrhythmia, psoriasis, diabetes, and cancers [1,6,7].

VLC-PUFAs are sensitive to oxidative stress and cellular responses, which can be triggered by day–night variations in salt concentrations in coastal waters of mangrove forests [3]. This may explain the evolutional development of the high level of antioxidant carotenoids in the cells of thraustochytrids. As pigmented microorganisms, thraustochytrids belong to the family of carotenogenic microorganisms that contain carotenoids in forms of astaxanthin, echinenone, canthaxanthin, and beta-carotene [8,9,10,11]. These molecules are important for human health because of their antioxidative properties [1,9]. Dietary carotenoids have beneficial properties delaying the onset of many diseases such as arteriosclerosis, cataracts, age-related macular degeneration, multiple sclerosis, cardiovascular diseases, and some kinds of cancer [12]. Currently, most commercially available carotenoids are manufactured through chemical synthesis. Nevertheless, there is a growing demand for naturally produced carotenoids due to increased bioactivity when compared to the synthetic equivalents [9,11].

Squalene, an intermediate of ergosterol synthesis, is widely used in the pharmaceutical industry for the delivery of vaccines, drugs, and other medicinal substances as well as in the cosmetic industry as a hydrating and antioxidant agent. It also improves the immune system and is used as a protective agent in cancer treatment [13,14]. The squalene market is currently growing and is expected to reach 4000 T with a value of USD 177 million by 2019 (www.marketsandmarkets.com). So far, deep-sea shark’s liver is the main source of squalene, producing 400–860 g/kg, or virgin olive oil, producing up to 5g/kg [14,15]. *Aurantiochytrium* species showed the highest concentration of squalene among thraustochytrids, accumulating up to 318 mg/g DW [1,16]. Other representatives of thraustochytrids showed a lower level of squalene production, 0.15–84 g/kg DW [17,18,19,20].

The aim of this paper is to assess the potential use of two new *Aurantiochytrium* strains as feedstock for the food, nutraceutical, and biofuel industries. Heterotrophic production of valuable biomolecules, such as ω3-PUFAs (DHA, EPA, and DPA), carotenoids, protein, carbohydrates, and squalene, was assessed intracellularly in biomass and in de-oiled cakes, and extracellularly, in supernatants and in extracellular polysaccharides (EPS) and recovered oil bodies (OBs). Alkalinization of collected cells to pH 12 represents a new and easy way to recover lipid-rich OBs in the form of an aqueous emulsion. This offers an easy strategy to produce DHA-rich lipids without an expensive extraction procedure.

## 2. Results and Discussions

### 2.1. Phylogenetic Analysis of Thraustochytrids

**Figure 1:**
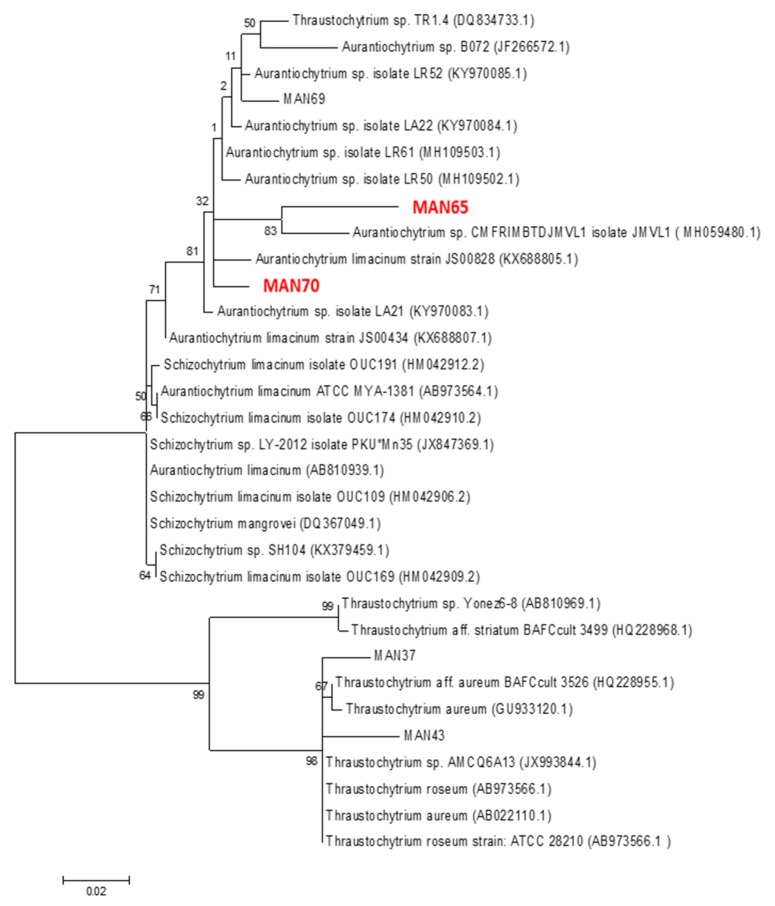
Phylogenetic analysis of thraustochytrids strains.

Isolated strains were identified phylogenetically using maximum parsimony and distance trees generated by using partial 18S rRNAs genes amplified with a set of primers for fungi/yeast described in [21] and were grouped into the genera *Aurantiochytrium* and designated as MAN65 and MAN70, GenBank accession numbers MH790117 and MH790119, respectively (Figure 1).

### 2.2. Biomass Production

**Figure 2:**
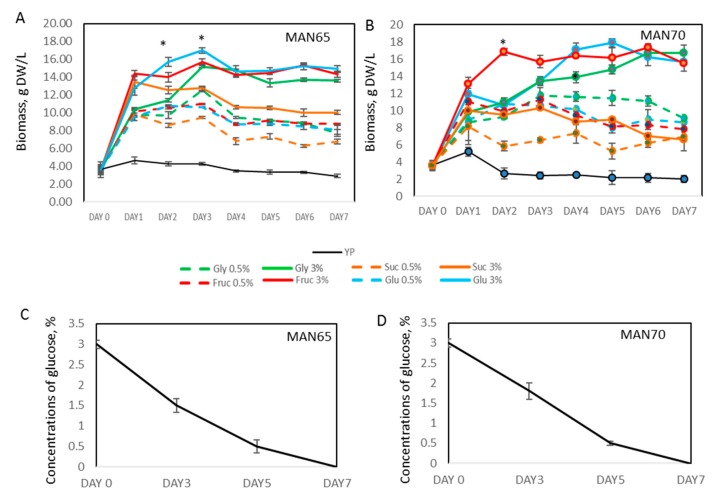
Growth of thraustochytrids cells in media supplemented with different carbon sources. (**A**,**B**): Growth rates. (**C**,**D**): Consumption rates of glucose molecules. Glu: glucose; Gly: glycerol; Fru: fructose; SUC: sucrose; * Significance levels: *p* < 0.05.

To evaluate the effect of the different carbon sources on the growth of two *Aurantiochytrium* strains, MAN65 and MAN70, growth media (YP) was supplemented with fructose, glucose, sucrose, and glycerol at concentrations of 0.5% and 3% (Figure 2). Both strains showed no significant growth in YP medium. Addition of glucose, fructose, or glycerol at concentrations of 0.5% and 3% resulted in up to 3- and 4.5-fold increase in cell growth by day 3, respectively, producing up to 18.1 gDW/L of biomass (*p* < 0.05), with productivities up to 5.7 gDW/L-day. Sucrose as a carbon source showed a low level of growth increase compared to other carbon sources, but higher than YP media alone. Efficient cell growth of thraustochytrid cells on glucose was associated with significant consumption of this sugar that showed no detectable levels at day 7 (Figure 2). Similarly, it is predicted that the growth of thraustochytrid cells in fructose or glycerol would have led to the complete consumption of the carbon source added initially.

The growth rates achieved in this study are higher than the published productivities for most of the characterized members of the thraustochytrids family [2,10,21,22]. However, higher growth rates, 26 gDW/L and 55 gDW/L, were documented when cultures were batch-fed with higher concentrations of the carbon source [23,24].

### 2.3. Intracellular Production of Lipids, Protein, Carbohydrates, Squalene, and Carotenoids

**Figure 3:**
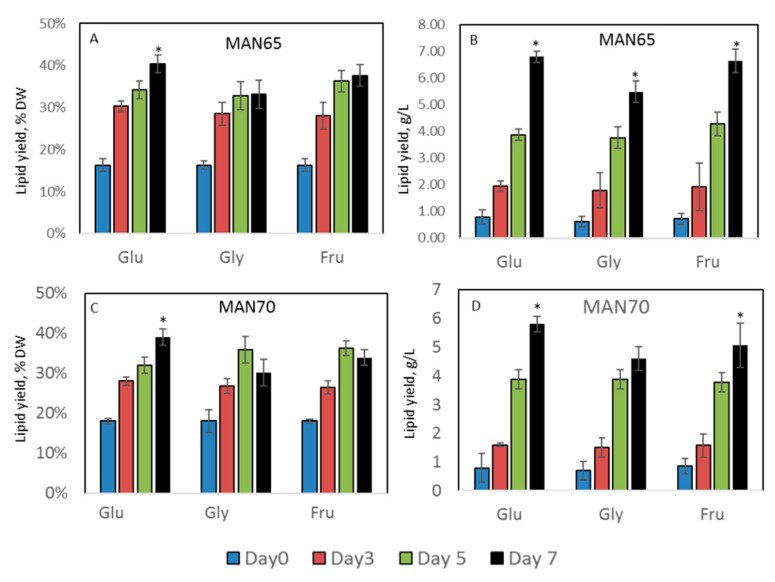
Lipid yields in thraustochytrids cells. (**A**,**B**): MAN65; **C**,**D**: MAN70. Glu: glucose; Gly: glycerol; Fru: fructose. * Significance levels: *p* < 0.05.

Lipid production in MAN65 and MAN70 cells grown in YP media supplemented with three carbon sources is shown in Figure 3 and Figure S2. The addition of 3% glucose, fructose, or glycerol led to an increase in accumulation of lipids, reaching up to 40% DW (6.8 g/L, *p* < 0.05) by day 7. 

In MAN65, the fatty acid methyl esters (FAMEs) composition varied depending on the carbon source used, but the proportions of saturated fatty acids (SAFAs) (up to 55% of total FAMEs) and PUFAs (up to 55% of total FAMEs) were roughly equivalent (Table 1). The fatty acid composition of MAN70, however, was dominated by PUFAs (up to 80% of total FAMEs). In both strains, SAFAs were mainly represented by palmitic acid, C16:0, at concentrations up to 119 mg/g DW which represents 77% of SAFAs (MAN65 grown on glucose). PUFAs were mainly represented by the ω3-containing fatty acids, docosahexaenoic acid, DHA, C22:6 at concentrations up to 157 mg/g DW which represents 78% of PUFAs (MAN70 grown on glucose) and docosapentaenoic acid, DPA, C22:5 (up to 32 mg/g DW representing 22% of PUFAs in MAN65 grown on glucose). Both strains showed only modest levels of EPA (up to 5.5 mg/g DW representing 14.7% of PUFAs in MAN65 grown in glycerol). Monounsaturated fatty acids (MUFAs) are mainly represented by oleic acid, C18:1. High concentration of PUFAs enriched with DHA (EPA:DPA:DHA as 1:5:21) is a signature for a large number of thraustochytrid representatives [2,5,8,21,22,24,25].

**Figure 4:**
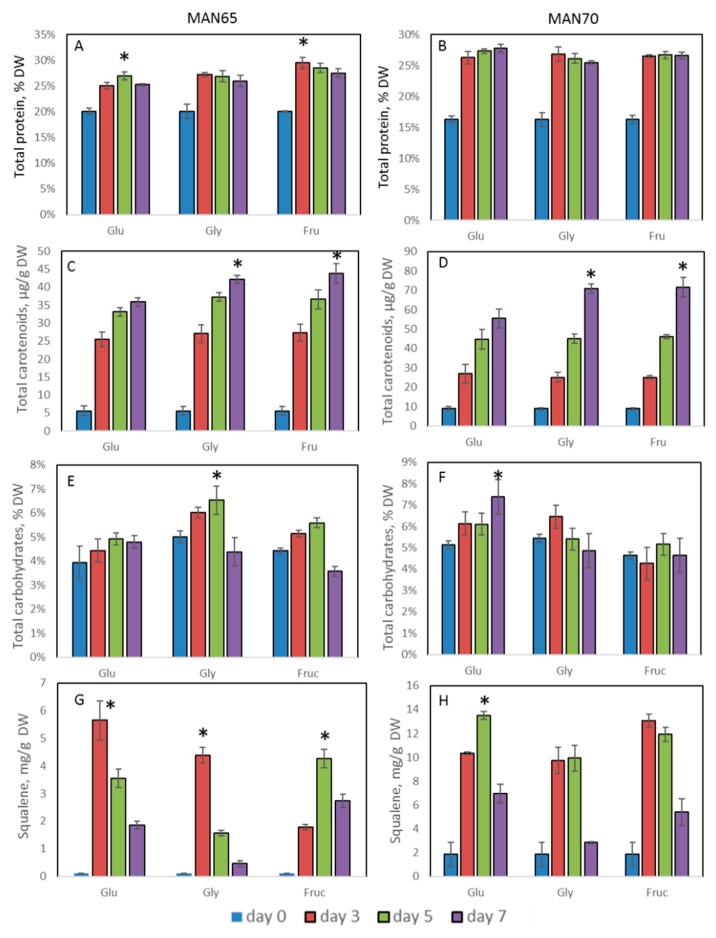
Concentrations of protein, carotenoids, carbohydrates, and squalene in thraustochytrids cells. (**A**,**B**): protein; (**C**,**D**): carotenoids; (**E**,**F**): carbohydrates: (**G**,**H**): squalene. (**A**,**C**,**E**,**G**): MAN65; (**B**,**D**,**F**,**H**): MAN70. Glu: glucose; Gly: glycerol; Fru: fructose. * Significance levels: *p* < 0.05.

Thraustochytrids have not been widely analyzed for protein production, however, growth on different carbon sources showed that these strains could produce up to 30% DW of crude proteins by day 3 (Figure 4A,B). These levels did not change significantly over the following four days. This amount of protein is lower than in soybeans (36–56% DW) and the cyanobacteria Spirulina (up to 70% DW) [26], but is, however, much higher than in aquatic plants, duckweed and Azolla, widely used as sources of protein for livestock [27]. To date, only a few publications related to the yield of protein in thraustochytrids are available; these reports claim production of up to 50% DW of protein, containing all essential and nonessential amino acids [5,28].

Both isolated strains showed coloration after growing in YP media supplemented with glycerol, glucose, and fructose: MAN65—yellow and MAN70—orange coloration (Figure S3). The levels of total carotenoids were increased up to 8-fold by day 7 (Figure 4C,D). The composition of four main carotenoids-astaxanthin, canthaxanthin, echinenone, and β-carotene-in thraustochytrids cells are shown in Table 2. Our data correlate with the level of the carotenoids isolated and characterized from other thraustochytrids species [9,10,11]. MAN70 showed a significantly higher production of total carotenoids than MAN65 (up to 70 µg/g DW), which also was obvious from the differences in their cells’ coloration.

Regardless of the source of carbon, the accumulation of total levels of carbohydrates in the strains MAN65 and MAN70 was generally low. The use of glycerol and collection at day 5 for MAN65 and glucose and collection at day 7 provided the best combinations for carbohydrate production (7.5% DW) (Figure 4E,F).

MAN65 and MAN70 strains grown on 3% glucose, fructose, and glycerol showed a wide range of squalene concentrations (between 1.5 and 13 mg/g DW, Figure 4G,H). In general, both strains showed an increase in squalene production after growth on carbon sources, compared to their starved stage, on day 0. MAN70 showed a higher level of squalene production than MAN65, accumulating up to 13 mg/g DW. This accumulation profile is not very different from data published for 132 analyzed isolates, which mostly showed squalene yields between 7.5 and 33 mg/g DW [17,20]. The highest concentrations of squalene were reported in *Aurantiochytrium* sp. 18W-13a with levels ranging from 171 to 198 mg/g DW and up to 318 mg/g DW in *Aurantiochytrium* sp. Yonez [1,16]. In general, *Schizochytrium* sp, showed lower levels of accumulation of squalene compared to *Aurantiochytrium* sp, even though the levels of squalene seem to vary with strain. The concentration of squalene was reported from 0.15 mg/g in three *Schizochytrium mangrovei* strains [17] to 84 mg/g DW in a *Schizochytrium* sp. CCTCC M209059b strain [18,19].

The kinetics of squalene accumulation in living cells are unclear, and as an intermediate product in ergosterol biosynthesis, its production and stability can be regulated by several internal and environmental factors. Under rapid growth and aerobic conditions, squalene concentration can be decreased because of its conversion to ergosterol by the enzyme squalene epoxidase [29]. Mutations of this enzyme or its inhibition by terbinafine in yeast *Saccharomyces cerevisiae* led to increased levels of squalene [1]. It was shown that in the *A. mangrovei* PQ6 strain, squalene was accumulated faster than the increase in lipids and it reached a maximum level after day 4 [17]. In *Schizochytrium* sp., the squalene content dropped from 439.98 mg/g to 0.88 mg/g during the lipid accumulation stage [19].

Thraustochytrid’s biomass after extraction of lipids (de-oiled cake) can be used as a source of various nutrients because of the presence of protein and carbohydrates. Table 3 shows that after lipid extraction which reduced lipid concentration from 29% DW to 2% DW in MAN65 and from 28% DW to 3 in MAN70, de-oiled cakes contained up to 28% DW of protein (280 mg/g DW) and 22% DW of carbohydrates (220 mg/g DW). De-oiled cakes have been the subject of numerous studies because of their utilization in various fields [30,31]. De-oiled microalgal biomass was directly used as a nutrient supplement for livestock and fish [32], as fertilizer [33], for biosorption [34], and for the production of valuable chemicals and biofuel via fermentation, thermochemical conversion, hydrothermal liquefaction, pyrolysis, or gasification [30].

### 2.4. Extracellular Production of Protein, Lipids, Carbohydrates, EPS, and Recovered Oil Bodies

**Figure 5:**
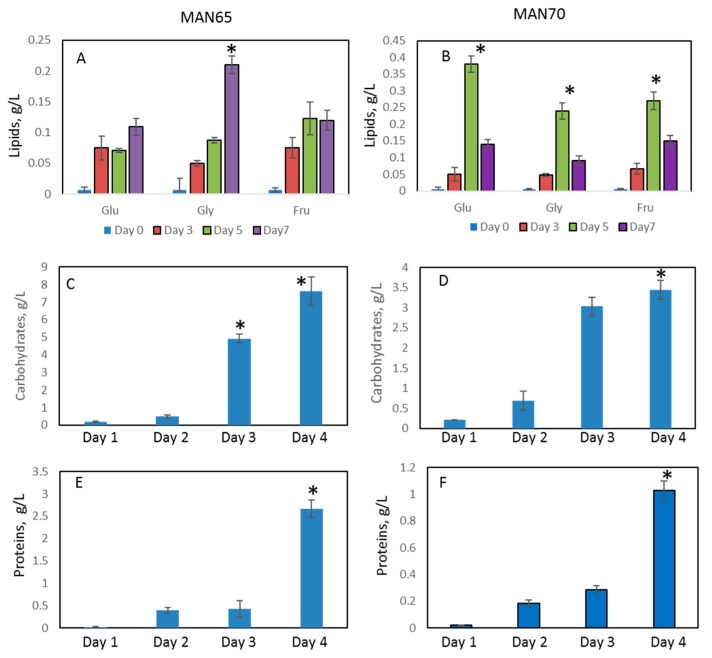
Accumulation of lipids, proteins, and carbohydrates in supernatants. (**A**,**B**): lipids; (**C**,**D**): carbohydrates; (**E**,**F**): proteins. (**A**,**C**,**E**): MAN65; B,D,F: MAN70. Glu: glucose; Gly: glycerol; Fru: fructose. * Significance levels: *p* < 0.05.

Supernatants of MAN65 and MAN70 grown on carbon sources were analyzed for the presence of lipids, protein, carbohydrates, and EPS (Figure 5). The concentration of lipids in supernatants increased up to 3.8-fold during cell growth and reached 0.2 g/L and 0.4 g/L for MAN65 and MAN70, respectively. Concentrations of carbohydrates in supernatants were analyzed only for cells grown in glycerol and were higher for MAN65 than for MAN70 (7.6 g/L and 3.5 g/L, respectively, *p* < 0.05). There was a significant (up to 130-fold) increase in protein levels in supernatants of MAN65 at the end of the experiment. Concentrations of protein were higher in MAN65 than in MAN70 (2.5 g/L and 1 g/L, respectively). No detectable levels of carotenoids and squalene were observed in supernatants of both thraustochytrid strains.

In supernatant, the presence of lipids, protein, and carbohydrates was detected as parts of EPS, the concentration of which was increased up to 63-fold after seven days of growth, reaching 6.3 g/L for MAN65 and 4.9 g/L for MAN70 (Table 4). Light microscopy examination of the grown cells showed the extensive production of EPS around the thraustochytrids cells as well as proteins and lipids incorporated in their scaffolds (Figure S4). Our data showed a higher concentration of EPS by day 7 than in previously published reports [35,36]. The production of extracellular EPS has been reported in various marine organisms, including plants, animals, diatoms, microalgae, and bacteria. These substances containing protein, carbohydrates, and lipids have a vast range of biotechnological applications [37,38,39,40]. The accumulation of EPS could confer biological benefits to the cells by protecting them from biotic and abiotic stresses and serve to reserve energy sources. It was shown that thraustochytrids cells could stay viable at least two days after desiccation [35]. EPS have been explored for various biotechnological applications, such as antitumor agents, anticoagulants, and wound dressings for eye and joint surgery. Apart from their medical applications, EPS are also important as emulsion stabilizers, flocculants, gelling agents and hydrating agents in cosmetics and pharmaceuticals [41,42,43].

**Figure 6:**
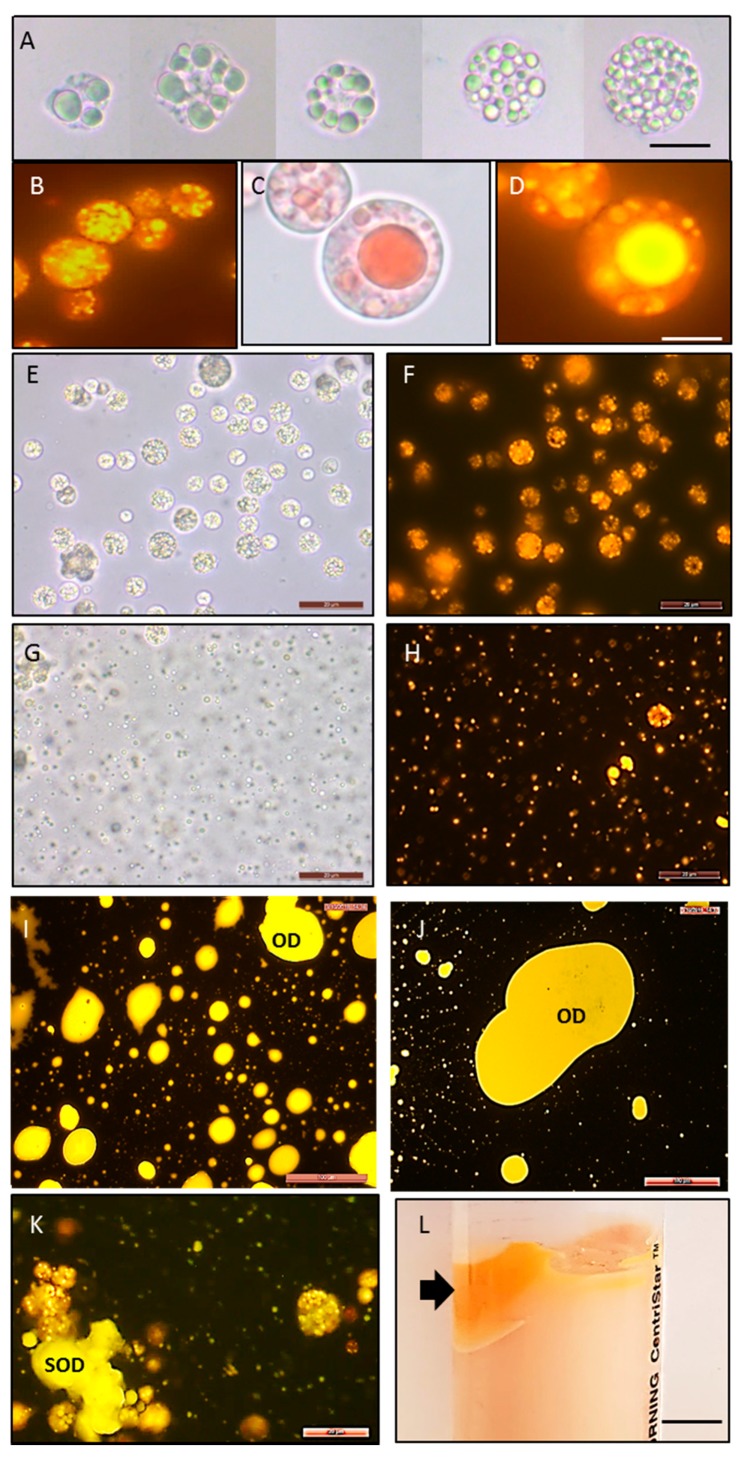
Recovery of oil bodies. (**A**–**D**): Oil bodies’ accumulation in cells; (**E**,**F**): cells before pH 12 treatment; (**G**–**H**): oil bodies 15 min after pH 12 treatment; (**I**–**K**: oil droplets and solidified oil droplets in the supernatant after 3–12 h of pH 12 treatment. (**L**): Accumulation of OBs (shown by arrow) after centrifugation; (**A**,**C**,**E**,**G**): bright-field microscopy; (**B**,**D**,**F**,**H**–**K**): fluorescent microscopy after staining lipids with Nile Red. OD: oil droplets; SOD: solidified oil droplets. Scale bars: (**A**–**H**, **K**) = 20 µm; (**I**,**J**) = 100 µm; (**L**) = 1 cm.

Microscopic analysis of thraustochytrid cells stained with Nile Red showed that intracellularly lipids accumulate within spherical bodies known as oil bodies (OBs). Figure 6 shows that OBs can be different in size, shape, and number, from many small droplets (appx 0.1–1 µm) to a single large droplet (14–20 µm), which can occupy up to 75% of the cell’s cytoplasm. To recover OBs from the cells, we adjusted the pH of cultured cells to 12, which led to the extensive rupture of the cell walls and the release of OBs into the supernatant. Microscopic analysis of the cells after 15 min of high pH treatment showed recovered OBs (Figure 6G,H). Longer treatment (3–12 h) led to the accumulation of liquid oil droplets (ODs) and solidified oil droplets (SODs) in the supernatant (Figure 6I–K). Centrifugation of the suspension led to the precipitation of cell debris and the concentration of this oil-rich biomass at the top of the solution (Figure 6L). After washing and drying, this biomass represented up to 34% of the original cells’ DW (cells before pH treatment) (Table 5). The extraction of lipids from oil-rich biomass unsurprisingly showed that they predominantly contained lipids (81% DW) and a low concentration of protein (up to 0.5% DW).

Resuspension of collected OBs collected after 15 min of treatment in water produced an emulsion which was analyzed for particle size distribution and electrical charge (zeta potential). The size of the particles in the emulsions at pH 7 revealed diameters between 100 nm and 600 nm with a predominant average diameter at 245 nm (a sharp peak, Figure S5). A much broader distribution of particle sizes was observed at pH 8, between 50 nm and 1 µm.

The zeta potential was analyzed to assess whether the OBs contained protein. Its value had changed from −57 mV at pH 8 to +4 mV at pH 2.5, with the point of zero charges (isoelectric point) being around pH 3 (Figure S6). This suggests proteins exist in suspension after the aqueous extraction procedure and cover the OBs [44]. The protein-coated fatty-acid-rich OBs were discovered in various plant and microalgal cells, and their characteristics were extensively studied due to their technological importance for the food industry [45,46]. The isoelectric point of OBs from thraustochytrids was lower than that observed in plant species (between 4 and 6) [44,47]. This can be explained by the reduced amount of proteins covering OBs which elucidates the production of free oil droplets in the supernatant (Figure 6I–K).

## 3. Materials and Methods

### 3.1. Strain Isolation and Phylogenetic Analysis

Thraustochytrids cells were collected from mangrove leaves and sediments (Victoria, Australia. GPS location: −38.265605 144.496448) (Figure S1). The collected samples were vortexed for 5 s and then plated on GPY media (10 g/L Tryptone, 5 g/L yeast extract, 30 g/L glucose in 50% seawater) containing a cocktail of antibiotics (Penicillin 50 µg/mL, Nystatin 10 µg/mL, Rifampicin 50 µg/mL, Streptomycin 50 µg/mL) according to Gupta et al. [48]. The isolated strains were identified phylogenetically using maximum parsimony and distance trees generated using partial 18S rRNAs genes amplified with a set of primers for fungi/yeast described in [48] and were grouped into the genera *Aurantiochytrium* and designated as MAN65 and MAN70, GenBank accession numbers MH790117 and MH790119, respectively.

### 3.2. Growth of Thraustochytrids

All isolates were grown in YP medium (yeast extract, 5 g/L and peptone, 10 g/L in 50% seawater) supplemented with glucose, glycerol, fructose, or sucrose (all from Sigma-Aldrich, Castle Hill, NSW, Australia) at the concentrations indicated in the results section. Cells were starved for 24 h prior to adding the different carbon sources by growing the cells on YP medium. Cell cultures were centrifuged at 6000 rpm for 10 min and washed twice with Milli-Q water. Cells’ dry weight was determined by filtering the cell suspension through a preweighed filter paper (Whatman GF/C, GE Healthcare Life Sciences, Maidstone, UK) and drying at 80 °C. Glucose, glycerol, fructose, and sucrose concentrations were analyzed by YSI 2900 Biochemistry Analyzer (YSI Incorporated Life Sciences, Yellow Springs, OH, USA). Fructose was analyzed using Sucrose/D-Fructose/D-Glucose Assay Kit from Megazyme (Wicklow, Ireland) (Cat N: K-SUFRG).

### 3.3. Biochemical Analysis

Lipid extraction and fatty acid methyl esters (FAMEs) analysis [27], carotenoid extraction [8] and analysis [10], and squalene analysis [17] were carried out as previously reported and described below.

#### 3.3.1. Lipid Extraction and FAMEs Analysis

Freeze-dried powdered biomass (25mg) was extracted in 4mL of chloroform/methanol (2:1, *v*:*v*) overnight. After centrifugation, the supernatant was transferred to a preweighed 5 mL glass vial. After removal of the organic phase under a stream of nitrogen, the content of total crude lipids in each sample was determined by weight difference. To determine the fatty acid composition of the lipids, 2.4mL of 6% H_2_SO_4_ in methanol was added to each vial, and the vial was sealed with a Teflon cap. After an acid-catalyzed transesterification at 80 °C for 3 h and further cooling, the FAMEs formed were extracted into hexane containing an internal standard (octacosane, 15mg/L) and analyzed directly by gas chromatography–mass spectrometry (GC-MS) using a standard mix (C4-C24, Supelco, Sigma-Aldrich, Castle Hill, NSW, Australia) containing 37 FAMEs for quantification of each fatty acid methyl ester.

#### 3.3.2. Carotenoid Extraction and Analysis

Freeze-dried powdered biomass (50 mg) was sequentially extracted in 1 mL of acetone and further centrifuged until the pellets were colorless. Petroleum ether (1 mL) and deionized water (1 mL) were added to the pooled acetone extract and further shaken and left until phase separation; organic phase was recovered and evaporated in nitrogen atmosphere. Analysis was carried out by high-performance liquid chromatography (HPLC).

#### 3.3.3. Squalene Extraction and Analysis

To 50 mg of biomass, 5 mL of solvent (chloroform/methanol 2:1) was added before vortexing for 1 min. The extract was left to shake overnight at 200 rpm and then centrifuged at 6000 rpm for 15 min. From the supernatant, 3 mL was transferred to a glass tube and dried in rotary vacuum (70 °C, 150 min). Samples were suspended in 1 mL of hexane and filtered into a vial before injection in a GC-MS.

#### 3.3.4. Total Carbohydrate Analysis

Total carbohydrates were extracted and analyzed according to Chandran et al. [49] using glucose as a standard. Each sample (25 mg) was ground with 1.25 mL of 2.5 N hydrochloric acid and boiled in a water bath for 3 h. After cooling at room temperature, solid sodium carbonate was used to neutralize the sample until the effervescence ceased. The sample volume was adjusted to 10 mL with distilled water and then centrifuged at 5000 rpm for 5 min. The supernatant was then collected for further analysis using the anthrone method.

#### 3.3.5. Total Protein Analysis

For total protein analysis, 1 mL of 0.1 M NaOH was added in 10 mg culture. The mixtures were vortexed for 1 min and left on ice for 1 h and vortexed again for 30 min. The mixture was centrifuged at 10,000 rpm in 5 min. The supernatant was filtered by using a 0.45 µm syringe filter. The protein quantification was measured by Bradford assay.

#### 3.3.6. Microscopy and Nile Red Staining

Microscopy studies and Nile Red staining were conducted, according to Miranda et al. [27].

Briefly, lipid staining with Nile Red was achieved by adding 5 µL of stain to a cell suspension and further incubating at 37 °C for 5min. The stained cells were then subjected to fluorescent microscopy analysis to observe the presence of lipid droplets using a Leica DM 2500 microscope with an attached camera Leica DFC 310 FX. Nile Red filter: excitation at 543 nm, emission 555–650 nm.

### 3.4. Harvesting and Characterization of EPS

EPS were harvested, according to Chang et al. [50]. Alcian Blue (1.0%) in 3% acetic acid, pH 2.5 was used to stain EPS. Lipids and protein within EPS were stained by Nile Red and Amido Black, respectively.

### 3.5. Oil Body Recovery

Cells were centrifuged at 6000× *g* for 10 min and washed and resuspended in Milli-Q water with pH 12 adjusted with 3M NaOH. The cells were then stirred for 15 min, 3 h, 6 h, 12 h and centrifuged at 3800× *g* for 20 min at 4 °C. The upper waxy layer was quantitatively transferred to a preweighed tube and dried overnight at 80 °C. This process was repeated three times to increase the yield of oil bodies. For lipid extraction, 5 mL of chloroform/methanol 2:1 was added to the tube which contained 50 mg oil bodies and left shaking overnight according to Miranda et al. [27]. Particle size distribution and zeta potential of oil bodies were conducted according to Nikiforidis et al. [45] using a Zetasizer Nano ZS.

### 3.6. Statistical Analysis

All experiments in this study were conducted in triplicate. All data are expressed as a mean ± standard deviation. The experimental data were subjected to one-way analysis of variance (ANOVA) as implemented in the GraphPad InStat 3 statistics platform. Tukey simultaneous tests were conducted to determine the statistical differences between treatments. To ascertain that the observed variations were statistically significant, the probability (P) values were determined. A 95% confidence level (*p* < 0.05) was applied for all analyses.

## 4. Conclusions

Two isolated *Aurantiochytrium* strains showed intracellular and extracellular production of valuable biomolecules, including an enriched concentration of PUFA/DHA lipids, carotenoids, squalene, carbohydrates and protein. The use of de-oiled cakes and nutrient-rich supernatants for additional recovery of added-value molecules makes their production more economical. Recovery of PUFA-rich oil bodies in the form of aqueous emulsion could be beneficial for food manufacturers, providing a new way to stabilize lipids against chemical or physical degradation during processing. All of this makes thraustochytrids an important alternative to microalgal and plant species currently dominating the food, pharmacological, nutraceutical, cosmetics, and biofuel industries.

## Figures and Tables

**Table 1:** FAME composition in MAN65 and MAN70 grown on carbon sources.

Strains/Carbon sources ^#^	FAME			Concentrations of FAMEs, mg/g DW								
	SAFA %	MUFA%	PUFA %	C15:0	C16:0	C17:0	C18:0	C18:1	C20:4	C20:5, EPA	C22:5, DPA	C22:6, DHA
MAN65_GLY	51.6 ± 7.5	2.8 ± 0.4	46.7 ± 7.2	26.6 ± 5.8	54.3 ± 6.7 *	9.1 ± 1.3	9.3 ± 2.3	5.5 ± 1.1	3.4 ± 1.1	5.5 ± 1.6	14.6 ± 3.3	66.2 ± 11.1 *
MAN65_GLU	43.7 ± 6.2	1.8 ± 0.5	54.5 ± 10.2	16.6 ± 9.8	119.4 ± 23.4 *	9.1 ± 1.8	9.6 ± 1.5	5.8 ± 1.2	3.0 ± 1.6	0.8 ± 1.1	31.6 ± 4.2	147 ± 18.7 *
MAN65_FRU	55.6 ± 4.7	3.7 ± 0.3	40.5 ± 4	8.9 ± 1.7	34.5 ± 10.0 *	3.4 ± 1.0	6.7 ± 0.6	3.6 ± 0.3	5.4 ± 0.9	5.8 ± 1.8	4.6 ± 0.10	33.6 ± 6.8 *
MAN70_GLY	18.5 ± 4.2	0.6 ± 0.2	80 ± 18.2	8.5 ± 1.5	8.2 ± 1.7 *	5.8 ± 1.3	9.0 ± 1.6	1.2 ± 0.2	0.8 ± 0.1	3.2 ± 0.6	29.7 ± 5.4	104.0 ± 22 *
MAN70_GLU	22.5 ± 3.3	0.65 ± 0.3	76 ± 17.3	19.2 ± 3.7	14.7 ± 2.4 *	9.8 ± 1.4	8.9 ± 2.7	1.2 ± 0.3	0.8 ± 0.2	1.0 ± 0.6	25.7 ± 5.0	157.5 ± 21.1 *
MAN70_FRU	21.6 ± 3.0	0.4 ± 0.3	78 ± 10.6	10.5 ± 7.7	14.7 ± 4.4 *	8.5 ± 1.5	6.0 ± 1.1	0.8 ± 0.3	0.5 ±0.3	1.8 ± 0.4	22.7 ± 4.4	123.0 ± 28.1 *

^#^ 5 days of growth; Glu: 3% glucose; FRU: 3% fructose; GLY: 3% glycerol. * Significance levels: *p* < 0.05.

**Table 2:** Composition of carotenoids.

Strains	Total ^#^	Carotenoids, %				Cells Color
		Ast, %	Canth, %	Echi, %	β-Car, %	
MAN70 ^#^	35.8 ± 2.1	7.5 ± 0.8	35.6 ± 10.1	33.0 ± 7.2	21.3 ± 8.2	O
MAN65 ^#^	55.2 ± 11.2	1.1 ± 0.5	36.6 ± 8.42	32.3 ± 10.2	30.7 ± 9.8	Y

^#^ after 3 days of growth in YP + 3% glucose; Ast: astaxanthin; Canth: canthaxanthin; Echi: echinenone; β-Car: β-carotene. Y: yellow; O: orange.

**Table 3:** Concentrations of lipids, proteins, and carbohydrates in de-oiled cakes.

Biomass	Lipid		Proteins		Carbohydrates	
	% DW	mg/g DW	% DW	mg/g DW	% DW	mg/g DW
MAN65 cells	29 ± 2.1	300 ± 27.0	22 ± 7.0	220 ± 17.5	9 ± 1.0	90 ± 8.2
MAN65 cake	2.1 ± 0.2	1.8 ± 0.1	28 ± 3.1	280 ± 18.2	16 ± 2.1	160 ± 17.0
MAN70 cells	28 ± 3.7	295 ± 32.1	16 ± 2.6	160 ± 12.4	17 ± 2.5	170 ± 12.2
MAN70 cake	3.2 ± 0.2	2.1 ± 0.7	21 ± 3.7	210 ± 11.5	22 ± 4.5	220 ± 13.3

**Table 4:** Concentration and composition of EPS in supernatants.

Thraustochytrids/Time/Carbone Source	EPS (g/L)	Carbohydrates, %	Proteins, %	Lipids, %
MAN65/ Day 0/ Glu	0.11 ± 0.1	14 ± 1.5	7 ± 0.5	28 ± 4.5
MAN65/ Day 7/ GLY	6.2 ± 0.5	6 ± 0.6	3 ± 0.6	18 ± 3.8
MAN65/ Day 7/ FRU	5.5 ± 0.3	7 ± 0.7	15 ± 1.4	31 ± 11.2
MAN65/ Day 7/ GLU	6.3 ± 1.2	6 ± 0.5	8 ± 1.7	13 ± 3.3
MAN70/ Day 0/Glu	0.1 ± 0.1	17 ± 2.4	11 ± 2.2	41 ± 6.5
MAN70/ Day 7/ GLY	4.0 ± 0.5	28 ± 5.5	14 ± 3.6	17 ± 4.4
MAN70/ Day/ 7 FRU	4.9 ± 0.3	14 ± 3.8	10 ± 0.5	21 ± 4.57
MAN70/ Day 7/ GLU	4.0 ± 0.5	12 ± 0.5	10 ± 0.5	32 ± 5.6

**Table 5:** Yields of oil bodies, lipids, and proteins.

pH 12 Treatment	Oil Body Yield, % DW	Lipid Yield, % Oil Body	Protein Yield, % Oil Body
15 min	28.1 ± 2.8	59.6 ± 5.5	0.22 ± 0.1
3 h	35.0 ± 2.2	67.4 ± 10.8	0.15 ± 0.1
6 h	20.0 ± 2.8	81.0 ± 12.5	0.26 ± 0.1
12 h	34.0 ± 2.2	82.1 ± 11.8	0.49 ± 0.1

## Supplementary Materials

The following are available online at https://www.mdpi.com/1660-3397/18/3/151/s1. Figure S1: A. Distribution of sampling sites in the mangrove habitats of Australia. B. Microscopic analysis of thraustochytrids strains. Thraustochytrid cells were grown in mangrove leaves (a, b); purified thraustochytrid cells (c) stained with Nile Red for lipid detection (d, yellow dots); thraustochytrid cells with accumulated carotenoids (e). a–c, e: bright-field images; (d) fluorescent microscopy. Scale bars: a: 5 cm; b: 1 cm; c–e: 20 µm. Figure S2. Microscopic analysis of thraustochytrids cells grown on different carbon sources. Bright-field and fluorescent microscopy of cells grown on seawater alone (SW) and YP media and carbon sources: 0.5% and 3% of glucose (Glu); glycerol (Gly); fructose (Fru); sucrose (Suc). Cells were stained for lipids with Nile Red. Scale bars: 20 µm. Figure S3. Carotenoids production in thraustochytrids cells. A: MAN65; B: MAN70. Figure S4. EPS accumulation in supernatant. A and B: EPS stained with 1% Alcian Blue; C: protein accumulation in EPS (staining with Amido Black); D: lipid accumulation in EPS (staining with Nile Red). Scale bars: A,C: 50 µM; B,D: 20 µM. A–C: bright-field microscopy. D: fluorescent microscopy. Figure S5: Particle size distribution in aqueous emulsion at pH 7 and 8. Figure S6: Dependence of the particle electrical charge (zeta potential) of extracted oil bodies on pH.
