# Supplementary material for: The Nutritional and Pharmacological Potential of New Australian Thraustochytrids Isolated from Mangrove Sediments
Source: Mar Drugs. 2020 Mar 6;18(3):151. doi: 10.3390/md18030151 (PMC7142457; doi:10.3390/md18030151)
Supplement: Supplementary file 1 [file marinedrugs-18-00151-s001.pdf]

SUPPLEMENTARY DATA

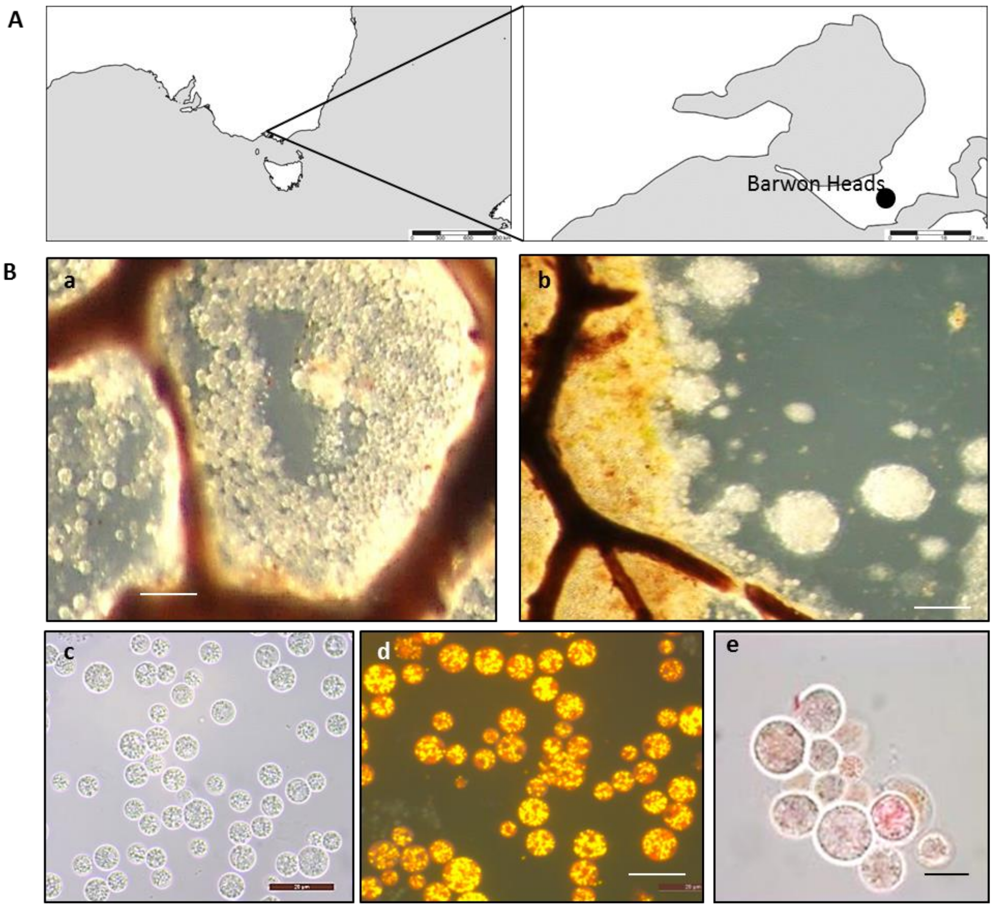


**Figure S1**

**Fig. S1**
A. Distribution of sampling sites in the mangrove habitats of Australia.
B. Microscopic analysis of thraustochytrids strains
Thraustochytrid cells were grown in mangrove leaves (a, b); Purified thraustochytrid cells (c) stained with Nile Red for lipid detection (d, yellow dots); Thraustochytrid cells with accumulated carotenoids (e).  a-c, e: bright-field images; (d) fluorescent microscopy. Scale bars: a: 5cm;  b: 1cm; c-e: 20 μm.

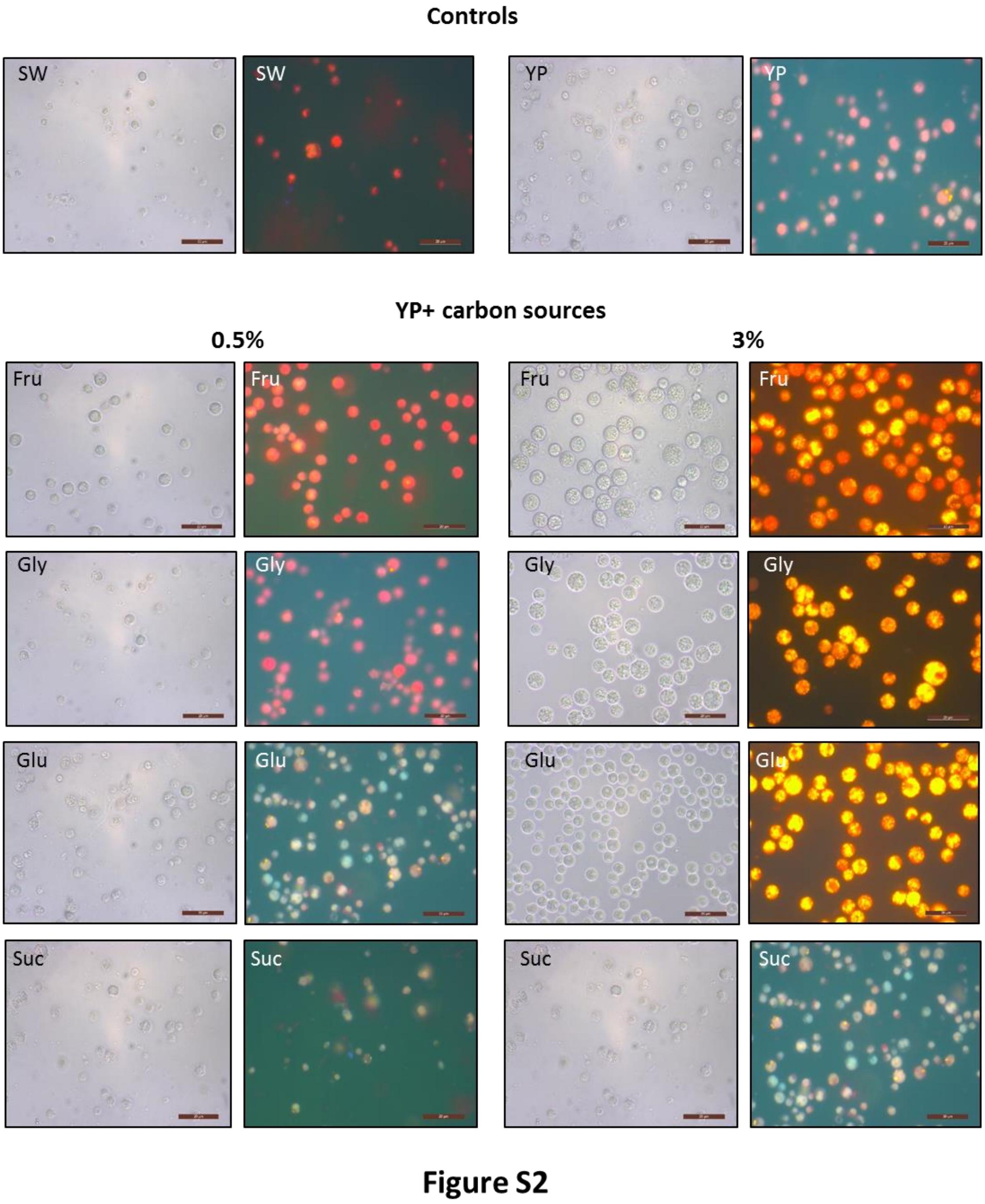


**Fig. S2.** Microscopic analysis of thraustochytrids cells grown on different carbon sources. Bright-field and fluorescent microscopy of cells grown on seawater alone (SW) and YP media and carbon sources: 0.5% and 3% of glucose (Glu); glycerol (Gly); fructose (Fru); sucrose (Suc). Cells were stained for lipids with Nile Red. Scale bars: 20 µm.

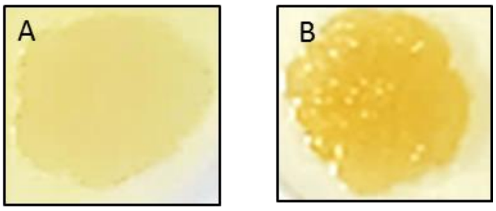


**Figure S3**

**Fig. S3.** Carotenoids production in thraustochytrids cells
A: MAN65; B: MAN70

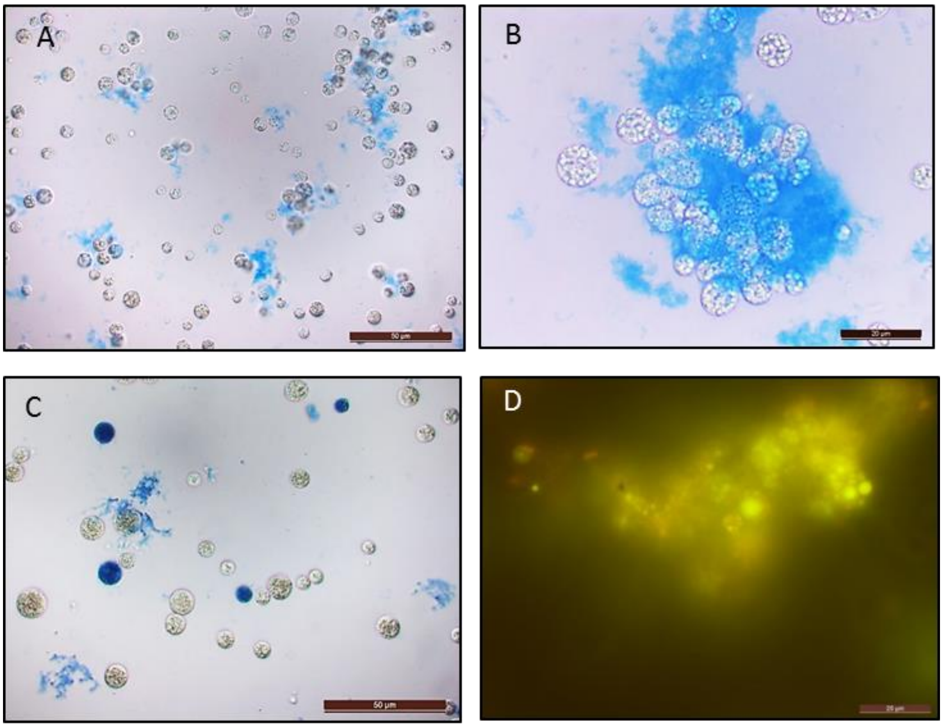


**Figure S4**

**Fig S4.** EPS accumulation in supernatant
A and B: EPS stained with 1% Alcian Blue; C: protein accumulation in EPS (staining with Amido Black); D: lipid accumulation in EPS (staining with Nile Red). Scale bars: A, C: 50 μM; B,D: 20 μM. A-C: bright-field microscopy. D: fluorescent microscopy.

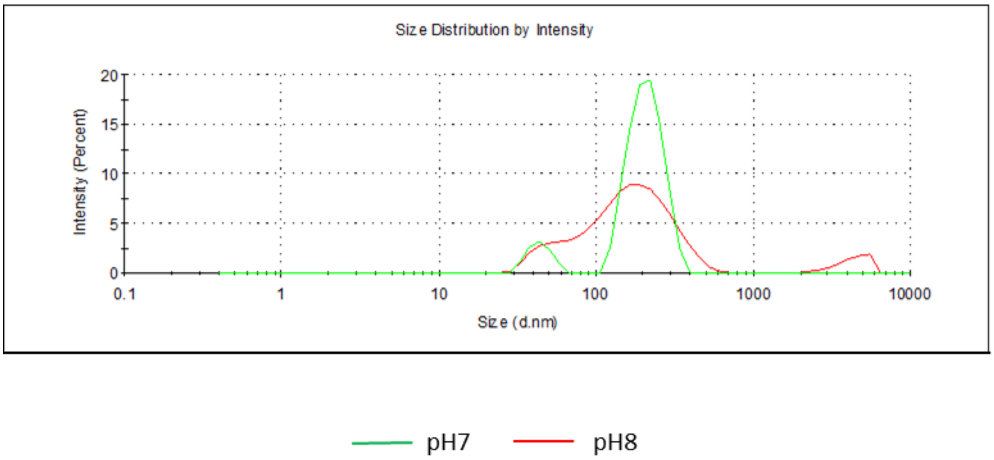


# Figure S5

**Fig. S5**: Particle size distribution in aqueous emulsion at pH 7 and 8.

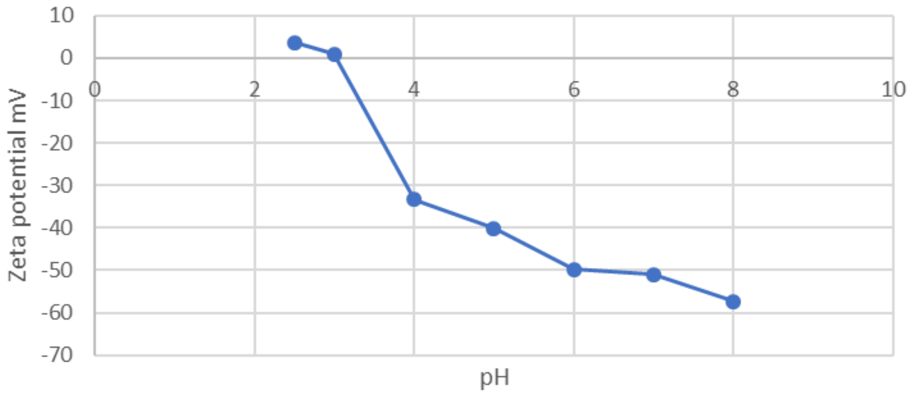


**Figure S6**

**Fig. S6:** Dependence of the particle electrical charge (zeta-potential) of extracted oil bodies on pH.
